# Allelic Variation in *PtGA20Ox* Associates with Growth and Wood Properties in *Populus* spp

**DOI:** 10.1371/journal.pone.0053116

**Published:** 2012-12-31

**Authors:** Jiaxing Tian, Qingzhang Du, Mengqi Chang, Deqiang Zhang

**Affiliations:** 1 National Engineering Laboratory for Tree Breeding, College of Biological Sciences and Technology, Beijing Forestry University, Beijing, China; 2 Key Laboratory of Genetics and Breeding in Forest Trees and Ornamental Plants, Ministry of Education, College of Biological Sciences and Technology, Beijing Forestry University, Beijing, China; University of Umeå, Sweden

## Abstract

*Populus tomentosa* is an economically important tree crop that produces wood for lumber, pulp, paper, and biofuels. Wood quality traits are likely to be strongly affected by the plant hormone gibberellic acid (GA), which regulates growth. *GA20Ox* encodes one of the major regulatory enzymes of GA biosynthesis and may therefore play a large role in growth and wood quality. Here, linkage disequilibrium (LD) studies were used to identify significant associations between single nucleotide polymorphisms (SNPs) within *PtGA20Ox* and growth and wood-quality traits of *P*. *tomentosa*. We isolated a full-length *GA20Ox* cDNA from *Populus tomentosa* by reverse transcription (RT)-PCR; this 1401 bp cDNA clone had an open reading frame of 1158 bp and encoded a protein of 385 amino acids. *PtGA20Ox* transcripts were maximally expressed in the mature xylem of vascular tissues, suggesting that *PtGA20Ox* is highly expressed and specifically associated with secondary xylem formation. Resequencing the *PtGA20Ox* locus of 36 individuals identified 55 SNPs, and the frequency of SNPs was 1/31 bp. The 29 most common SNPs (frequency>0.1) were genotyped in an association population (426 individuals) that was also phenotyped for key growth and wood quality traits. LD did not extend over the entire gene (*r* ^2^<0.1, within 500 bp), demonstrating that a candidate-gene-based LD approach may the best way to understand the molecular basis underlying quantitative variation in this species. SNP- and haplotype-based association analyses indicated that four SNPs (false discovery rate *Q*<0.05) and 14 haplotypes (*P*<0.05) were significantly associated with growth and wood properties. The phenotypic variance explained by each SNP ranged from 3.44% to 14.47%. The SNP markers identified in this study can be applied to breeding programs for the improvement of growth and wood-property traits by marker-assisted selection.

## Introduction

Gibberellins (GAs) are phytohormones that regulate a wide range of growth and developmental processes in plants, including seed germination, leaf expansion, stem elongation, and the development of flowers, seeds, and fruit [1], [2]. GAs also play an important role in promoting the formation of fiber in trees and ultimately in determining the quality of wood [3]. In higher plants, GAs are synthesized through a complex pathway in which GA20-oxidase (GA20Ox) is one of the major regulatory enzymes [4]–[6]. Previous studies have demonstrated that the expression of GA20-oxidase genes from various species can modify endogenous GA levels and in turn enhance plant growth [5], [7], [8]. Consequently, it is crucial to enhance our understanding of the role of GA20Ox in regulating growth and wood fiber properties to effectively manipulate wood biosynthesis in trees.

One of the main goals of forest tree breeding programs is to increase the quantity and quality of wood products. Marker-assisted selection (MAS) is a useful tool in tree breeding for reducing breeding cycles and increasing selection accuracy, particularly for wood properties [9]. Before MAS can be applied to tree breeding, markers that are significantly associated with target traits need to be identified. Association studies are powerful methods for identifying markers that are significantly linked to traits in natural or breeding populations [10]. DNA markers that are commonly used in association studies include single nucleotide polymorphisms (SNPs) and insertions or deletions (InDels) of the DNA sequence [11]. SNP-based linkage disequilibrium (LD) mapping provides another strategy for MAS in forest trees [12]. In contrast to traditional linkage analysis, LD mapping can be readily applied to natural or breeding populations of unrelated individuals to identify marker-trait associations. For example, Thumma *et al*. [13] discovered polymorphisms in the *cinnamoyl-CoA reductase* (*CCR*) gene that were associated with microfibril angle in *Eucalyptus nitens*. Similarly, 13 SNPs of five xylem genes associated with microfibril angle, cellulose, pulp yield, and total lignin were identified in *E. nitens* [14]. In addition, Wegrzyn *et al*. [15] found 27 highly significant, unique, single-marker associations (false discovery rate *Q*<0.10) across 40 candidate genes in three composite traits including the lignin content, syringyl to guaiacyl ratio, and C6 sugars in black cottonwood (*Populus trichocarpa*). Previous studies have demonstrated that LD mapping can be used to identify alleles associated with quantitative traits, suggesting that this new approach could be particularly useful in forest tree breeding programs.

Chinese white poplar (*P. tomentosa* Carr.) belongs to the section *Populus* in the genus *Populus*. This species is one of the main commercial trees for timber production in China and plays an important role in ecological and environmental protection along the Yellow River [16]. With the development of targeted cultivation of industrial commercial forests, new varieties of poplar must be fast-growing and produce high-quality wood. Therefore, methods to improve the growth and wood quality of *P. tomentosa* are essential. To this end, association studies of SNPs associated with growth and wood properties of *P. tomentosa* are important to improve the growth and wood quality of *P. tomentosa* by MAS breeding programs. Population structure is the leading cause of false positives in genetic association studies [17]. The presence of population structure leads to bias because subgroups of relatives tend to share more markers and gene alleles genome-wide than a pair of individuals drawn at random from the population [18]. Therefore, correction for the confounding effects of population structure present in plant populations is essential in association mapping. Huang [19] was the first to provide climatic regionalization in the distribution zones of *P. tomentosa* and showed that the three climatic zones can be treated as genetic regions. In addition, the structure of the natural populations has also been explored by Du et al [20]; we used this information on population structure in our association analysis in this study. Because the gene encoding GA20-oxidase, one of the major regulatory enzymes in GA synthesis, likely serves a key role in the regulation of growth and development [5], [7], [8], it can be used as a model to address the significance of allelic variation linked to growth and wood-quality traits in trees. Here, we report the identification and characterization of the gene encoding GA20Ox, *PtGA20Ox*, from a mature xylem cDNA library of *P*. *tomentosa*. Real-time PCR analysis revealed that this gene may be involved in wood formation and may be up-regulated by GA treatment in trees. Several common SNPs and their haplotypes in *PtGA20Ox* were identified for associations with growth and wood properties while accounting for population structure. The selected SNPs were being investigated further to test whether RNA transcript accumulation varies among the different genotypes that showed significant associations.

## Results

### Phenotypic Data Distribution and Correlations

To quantify traits for association mapping, we measured growth and wood-quality properties in our association population of 426 individuals (see Methods). In the association population, all 10 wood-quality and growth traits have abundant phenotypic variation; for example, fiber length, fiber width and volume values ranged from 0.866 to 1.512 mm (mean value of 1.170 mm), from 1.6984 to 33.503 µm (average of 23.160 µm), and from 0.037 to 3.022 m^3^ (mean 0.602 m^3^), respectively. The maximum tree height (22.50 m) was approximately eight times higher than that of the minimum tree (2.90 m), and the average height was 14.61 m. Descriptive statistics of the trait distributions are presented in Table S1. As expected, the frequency distributions for each trait measured in the association population followed an approximately normal distribution (data not shown).

The growth and wood-quality traits in the association population showed significant correlations (Table S2). For example, fiber length was positively correlated with fiber width (*P*<0.01). In addition, significant positive pairwise correlation was observed between tree diameter and volume (*P*<0.01). The details of the phenotypic correlations among these traits in the association population are shown in Table S2.

### Isolation of a *PtGA20Ox* Gene from *P. tomentosa*

**Figure 1:**
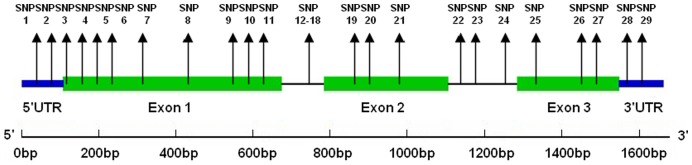
Genomic organization of *PtGA20Ox*. Exons are shown as boxes and introns as lines. Positions of common SNP markers are shown as vertical lines.

To identify *GA20Ox* SNPs, we first isolated the *GA20Ox* locus. A full-length cDNA encoding GA20Ox was isolated from a cDNA library prepared from the mature xylem zone of *P*. *tomentosa* by reverse transcription (RT)-PCR. The cDNA clone is 1401 bp in length and has an open reading frame of 1158 bp, including 126 bp of 5′ untranslated region (UTR) and 117 bp of 3′ UTR. Alignment of the full-length cDNA sequence with the genomic sequence showed that *PtGA20Ox* is composed of two introns and three exons (Figure 1). The deduced protein sequence of *PtGA20Ox* revealed a protein of 385 amino acids with an estimated molecular mass of 44.0 kD and a pI of 8.38. A BLASTP search with PtGA20Ox as the query sequence revealed that the PtGA20Ox protein shares 65.8% identity with the *Arabidopsis* GA20Ox.

### Expression Analysis of *PtGA20Ox*

**Figure 2:**
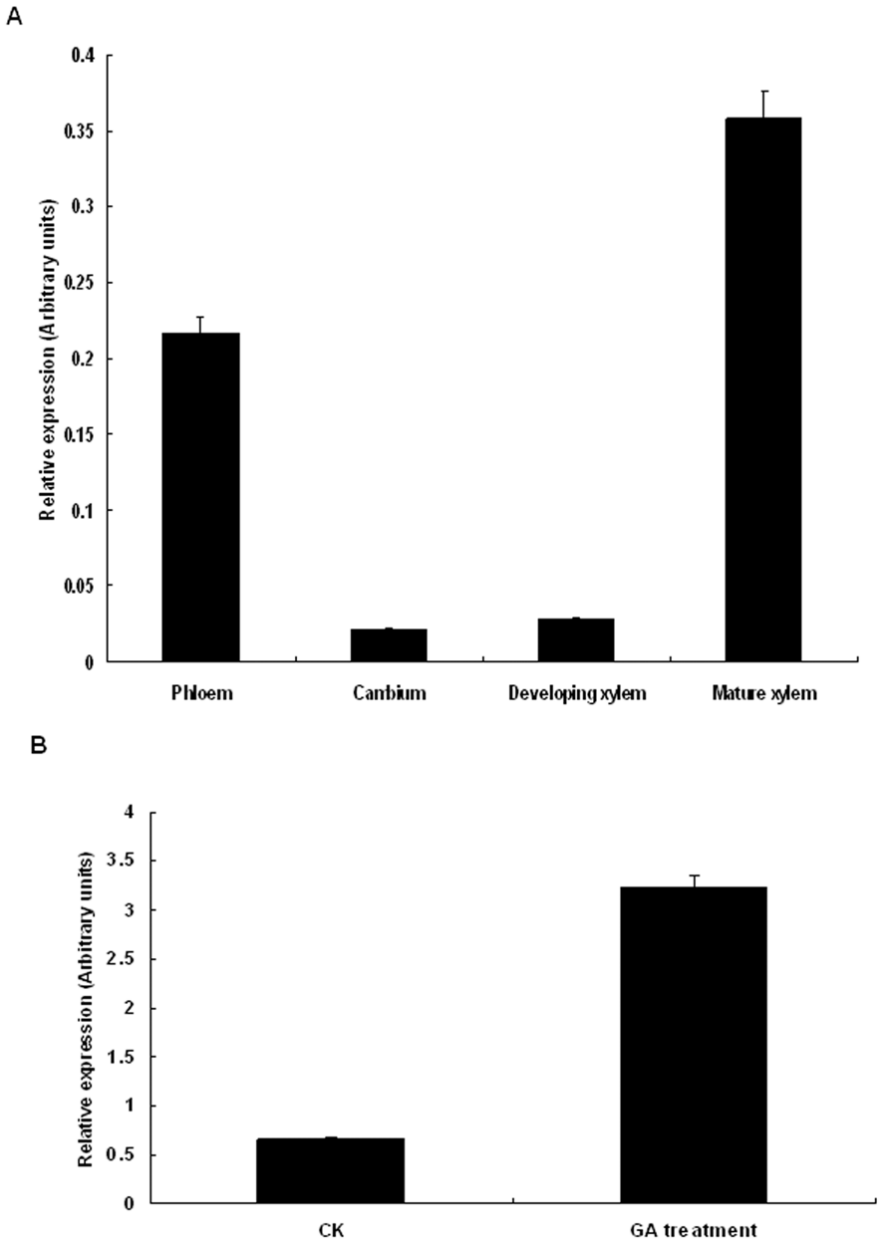
Relative transcript levels of *PtGA20Ox*. (A) Relative transcript levels of *PtGA20Ox* in various poplar vascular tissues. (B) Relative transcript levels of *PtGA20Ox* before and after GA treatment.

Using gene-specific primer and *Actin* as an internal control, real-time quantitative PCR was used to perform transcript profiling of *PtGA20Ox* mRNA in various poplar vascular tissues: phloem, cambium, developing xylem, and mature xylem. *PtGA20Ox* was preferentially expressed in mature xylem (Figure 2A); consistent with that its full-length cDNA was originally isolated from a cDNA library prepared from the mature xylem zone of *P. tomentosa*. By contrast, relatively lower levels of *PtGA20Ox* mRNA were detectable in the primary tissues of the cambium. Thus, *PtGA20Ox* appears to be a highly expressed gene specifically associated with secondary xylem formation. We further tested whether *PtGA20Ox* was inducible by treatment with GA (Figure 2B). The expression of *PtGA20Ox* after GA treatment was about five times higher than the expression of the control, suggesting that *PtGA20Ox* is up-regulated by GA.

### SNP Diversity and Genotyping

To identify polymorphisms for association mapping, we re-sequenced the *PtGA20Ox* region in 36 unrelated individuals from the association population. An approximately 1693 bp genomic region of *PtGA20Ox*, including 126 bp of 5′ UTR, 1158 bp of coding regions, 292 bp of intron, and 117 bp of 3′ UTR, was amplified and sequenced from 36 unrelated individuals, representing almost the entire natural range of *P*. *tomentosa*. Table 1 summarizes the statistical analysis of nucleotide polymorphisms (excluding indels) over different regions of *PtGA20Ox*. Across the samples, 55 SNPs were detected in the entire gene at a frequency of approximately one SNP every 31 bp (Table 1). Thirty of these SNPs were found in exons, of which 20, 9, and 1 variants were categorized as silent, missense, and nonsense mutations, respectively (Table 1). Altogether, 29 of 55 SNPs (53%) were considered common (frequency>0.10). In general, the *PtGA20Ox* locus has high nucleotide diversity (π_T_), where π_T_ = 0.00988 and θ_w = _0.00797 (Table 1). More specifically, estimates of nucleotide diversity for the different gene regions ranged from 0.00594 (exon 2) to 0.03478 (intron 1), and θ_w_ varied between 0.00442 (5′ UTR) and 0.02173 (intron 1). Within coding regions, the value of non-synonymous nucleotide substitutions (π_nonsyn_) was markedly lower than π_syn_, with a π_nonsyn_/π_syn_ ratio of 0.27, suggesting that diversity at the non-synonymous sites of exon regions resulted from strong purifying selection (Table 1). The 29 common SNPs were successfully genotyped across 426 individuals in the association population using locked nucleic acid (LNA) technology.

**Table 1:** Nucleotide polymorphisms at the *PtGA20Ox* locus.

Region	No. of bp	No. of polymorphic sites	Percentage polymorphism	Nucleotide diversity	
				π	θ_w_
5′ UTR	126	2	1.59	0.00936	0.00442
Exon 1	569	15	2.64	0.00865	0.00636
Synonymous	122.30	9	7.36	0.01901	0.01901
Non-synonymous	444.70	6	1.35	0.00583	0.00325
Intron 1	111	10	9.01	0.03478	0.02173
Exon 2	322	9	2.80	0.00594	0.00674
Synonymous	69.70	8	11.48	0.02587	0.02768
Non-synonymous	251.30	1	0.40	0.00043	0.00096
Intron 2	181	6	3.31	0.01012	0.00851
Exon 3	267	6	2.25	0.00655	0.00542
Synonymous	59.19	3	5.07	0.00458	0.01222
Non-synonymous	204.81	3	1.46	0.00721	0.00353
3′ UTR	117	7	5.98	0.01081	0.01455
Total silent^a^	761.19	45	5.91	0.01610	0.01426
Synonymous	252.19	20	7.93	0.01745	0.01912
Non-synonymous	902.81	10	1.11	0.00463	0.00267
Total *PtGA20Ox* b	1693	55	3.25	0.00988	0.00797

a Total silent = synonymous plus silent sites.

b Total *PtGA20Ox* = silent sites plus non-synonymous sites.

Regions containing indels were excluded from the calculations.

### Linkage Disequilibrium

**Figure 3:**
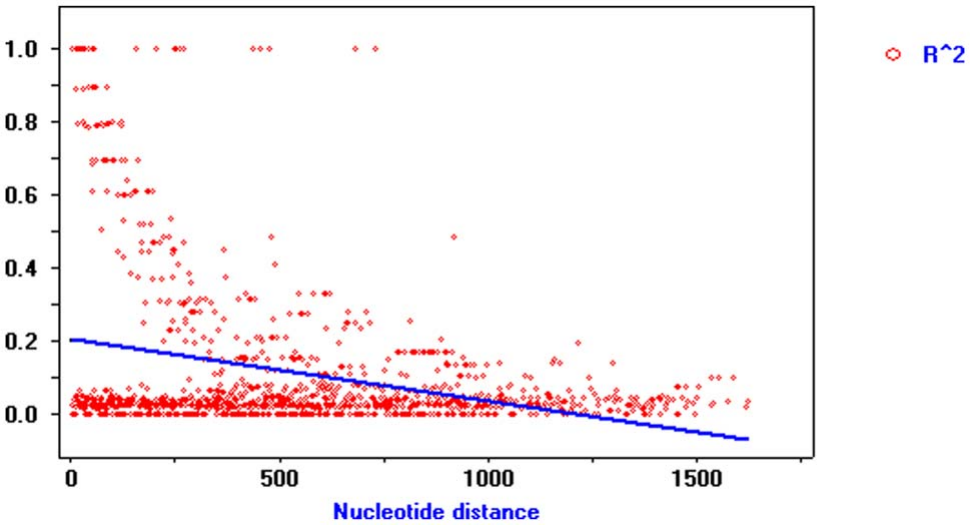
Decay of linkage disequilibrium within *PtGA20Ox*. Pairwise correlations between SNPs are plotted against the physical distance between the SNPs in base pairs. The straight line describes the least-squares fit of *r* ^2^ (Er^2^) to its expectation. Linkage disequilibrium decays drastically within 500 bp.

**Figure 4:**
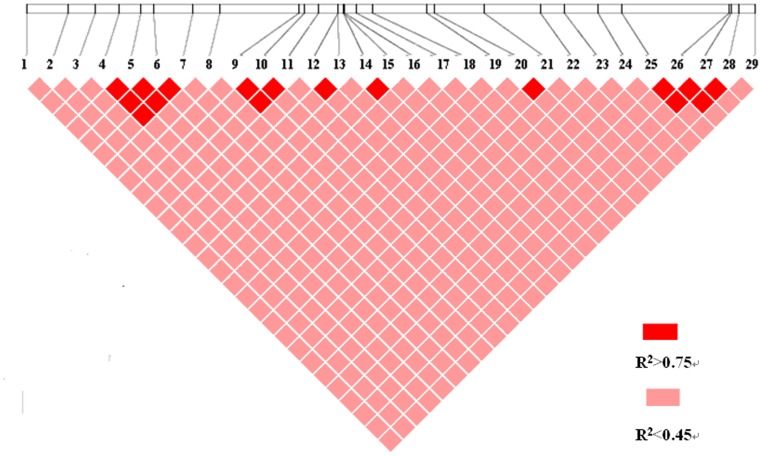
Pairwise linkage disequilibrium (*r^2^*) between SNP markers. The common genotyped SNPs are shown on a schematic of *PtGA20Ox* and the pairwise r^2^ values are shown by color coding in the matrix below.

The pattern of the squared allelic correlation coefficient (*r* ^2^) with base-pair distance within the *PtGA20Ox* gene illustrated rapid LD decay in the *P*. *tomentosa* population (Figure 3), with *r* ^2^ values dropping to 0.1 within 500 bp, indicating that the LD of the SNPs within this gene did not extend over the entire gene region. LD analysis using genotype data of 29 SNPs from 426 individuals in the association population (Figure 4) revealed three distinct haplotype blocks within the *PtGA20Ox* gene, from SNP4 to 7, SNP9 to 11, and SNP25 to 28. Within each block, LD between the SNPs was high (*r* ^2^>0.75), whereas LD was low between the three haplotype blocks (*r* ^2^<0.3) (Figure 4).

### Marker-trait Association and Haplotype Analysis

Associations between 29 SNPs and 10 growth and wood-quality traits were tested by comparing results from a general linear model (GLM) and a mixed linear model (MLM) in TASSEL version 2.1 software. The number of significant markers (*P*<0.05) was 15 using GLM but fell to 13 with MLM (Table S3). After a qFDR (false discovery rate) test, the number of significant associations of SNPs (*Q*<0.05) with growth and wood-property traits was reduced to seven. This analysis revealed that four SNP markers (SNP10, SNP19, SNP22, and SNP29) were significantly associated with five traits, including fiber length, fiber width, microfibril angle, holocellulose content, and tree height (Table 2). These associations were identified in exonic, intronic, and 3′ UTR regions of *PtGA20Ox*. Of these markers, SNP10, a missense mutation in exon 1 resulting in an encoded amino acid change from Asn to Lys, was significantly associated with multiple traits, i.e., fiber length, fiber width, holocellulose content, and tree height; SNP19 in exon 2, a synonymous mutation, was associated with fiber length, SNP22 in intron 2 was associated with fiber width, and SNP29 from 3′ UTR was closely linked to microfibril angle. Altogether, these SNP associations explained a small proportion of the phenotypic variance, with the individual effects ranging from 3.44% to 14.47%.

**Table 2:** SNP markers significantly associated with growth and wood-property traits using the mixed linear model (MLM).

Trait	Marker	Position	*P*- value	*Q-*value	R^2^	F_ST_
Fiber length	SNP10	Exon 1	8.72×10^−14^	1.26×10^−11^	14.47%	0.0023
	SNP19	Exon 2	9.94×10^−5^	0.0048	4.66%	0.0001
Fiber width	SNP10	Exon 1	4.17×10^−9^	3.02×10^−7^	10.20%	0.0023
	SNP22	Intron 2	0.0005	0.0160	4.13%	0.0016
Microfibril angle	SNP29	3′UTR	0.0003	0.0091	4.36%	0.0002
Holocellulose content	SNP10	Exon 1	5.61×10^−5^	0.0033	5.78%	0.0023
Tree height	SNP10	Exon 1	0.0010	0.0287	3.44%	0.0023

*P*-value = the significant level for association (the significance is *P*≤0.05), R^2^ = percentage of the phenotypic variance explained, *Q*-value = a correction for multiple testing [false discovery rate FDR (*Q*) ≤0.05], F_ST_ = variation due to differentiation among subpopulations.

Most of the associations were consistent with modes of gene action other than codominance (Table 3). For example, heterozygotes for SNP10 had shorter fiber length on average than either homozygote class (1.2804 µm for AA, 1.1688 µm for AG, 1.2167 µm for GG). Three of the seven marker-trait pairs for which dominance and additive effects could be calculated were consistent with over- or underdominance (|d/a|>1.25). The remaining four marker-trait pairs were split between modes of gene action that were partially to fully dominant (0.50<|d/a|<1.25, *n* = 2) or codominant (|d/a|≤0.5, *n* = 2). Differences in microfibril angle among the three genotypes of SNP29 were significant, indicating that the pattern of gene action was consistent with additive effects.

**Table 3:** List of marker effects for significant marker-trait pairs.

Trait	SNP	2a^1^	d^2^	d/a	2a/s_p_ 3	Frequency^4^		a^5^
Fiber length	SNP 10	0.0637	−0.0797	−2.5024	0.7611	0.48	(G)	0.0192
	SNP19	0.0392	−0.0114	−0.5816	0.4683	0.49	(T)	0.0152
Fiber width	SNP10	3.6247	−0.8306	−0.4583	1.8285	0.48	(G)	−0.5954
	SNP22	3.9557	−2.5851	−1.3070	1.9955	0.49	(T)	0.2697
Microfiber angle	SNP29	8.2694	1.3075	0.3162	1.8279	0.46	(C)	6.2997
Holocellulose	SNP10	7.7360	2.0023	0.5177	0.7192	0.48	(G)	−2.5755
Tree height	SNP10	1.4100	−2.0458	−2.9018	0.4905	0.48	(G)	0.5098

1 Calculated as the difference between the phenotypic means observed within each homozygous class (2a = |G_BB_–G_bb_|, where G_ij_ is the trait mean in the ijth genotypic class).

2 Calculated as the difference between the phenotypic mean observed within the heterozygous class and the average phenotypic mean across both homozygous classes [d = G_Bb_−0.5(G_BB_+G_bb_), where G_ij_ is the trait mean in the ijth genotypic class].

3 s_p_, standard deviation of the phenotypic trait under consideration.

4 Allele frequency of either the derived or minor allele. Single nucleotide polymorphism (SNP) alleles corresponding to the frequency listed are given in parentheses.

5 The additive effect was calculated as a = p_B_(G_BB_)+p_b_(G_Bb_)–G, where G is the overall trait mean, G_ij_ is the trait mean in the ijth genotypic class, and p_i_ is the frequency of the ith marker allele. These values were always calculated with respect to the minor allele.

We used haplotype trend regression (HTR) to identify significant haplotypes associated with growth and wood-quality traits from the regions surrounding the significant SNPs. We found 14 common haplotypes (frequency>1%) associated with growth and wood-quality traits (Table 4). Among these, three haplotypes from SNP9–11 were associated with fiber length, two haplotypes from SNP20–22 and three haplotypes from SNP10–12 were associated with fiber width, and six haplotypes from SNP8–10 were associated with tree height. The proportion of phenotypic variation explained by these haplotypes, which originated from exon 1, exon 2, intron 2, and 3′UTR, ranged from 3.98% to 8.37%.

**Table 4:** Haplotypes significantly associated with growth and wood-property traits.

Trait	*P-*value	R^2^	Haplotype	Frequency
Fiber length	7.93×10^−6^	8.37%	SNPs 9–11	
			C-A-C	0.492
			A-G-T	0.469
			A-A-T	0.025
Fiber width	0.0018	4.05%	SNPs 20–22	
			T-G-A	0.495
			C-A-T	0.495
	6.61×10^−6^	7.62%	SNPs 10–12	
			A-A-C	0.492
			G-T-A	0.491
			A-A-A	0.014
Tree height	0.003	3.98%	SNPs 8–10	
			T-C-A	0.247
			C-C-A	0.247
			T-A-G	0.237
			C-A-G	0.237
			T-A-A	0.014
			C-A-A	0.014

*P* = the significance level for haplotype-based association (*P*≤0.05); R^2^ = percentage of the phenotypic variance explained.

### Transcript Analysis of SNP Genotypes

**Figure 5:**
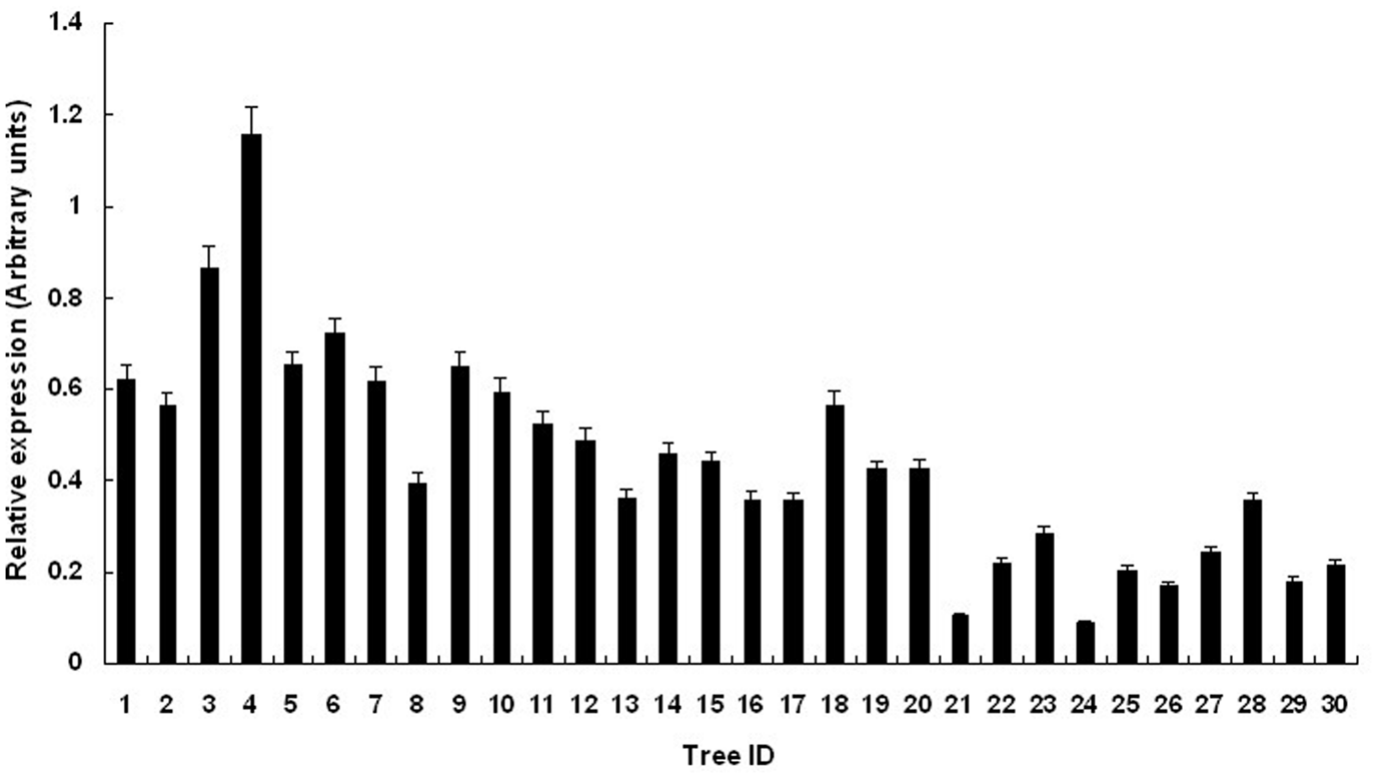
Expression levels of three genotypic classes for SNP10. 1–10 represent the AA group, 11–20 represent the AG group, and 21–30 represent the GG group.

To determine whether *PtGA20Ox* expression was altered in the different genotypic classes, transcript levels of the four significantly associated SNPs were compared using real-time quantitative PCR with gene-specific primers. The assays used secondary xylem from 20-year-old trees to quantify mRNA levels in 30 trees (10 trees for each genotype) of the association population. Among the four SNPs tested, only SNP10 exhibited significant differences in the transcript levels among the three genotypes (Figure 5). The highest *PtGA20Ox* mRNA levels were found in the AA group, followed by the AG group, and the transcript levels of the GG group were lowest. The mean relative expression levels of mRNA products for the AA, AG, and GG groups were 0.6832, 0.4407, and 0.2067, respectively. These results demonstrated that the transcript level of the AA group was about 1.55 times that of the AG group and 3.31 times that of the GG group.

## Discussion

### Analysis of Statistical Models for Association

An appropriate statistical model is necessary for phenotype-genotype associations to avoid false positive or spurious associations that arise from population and family structure [21]. One widely used approach, the general linear model (GLM) was first implemented in TASSEL (Trait Analysis by aSSociation, Evolution and Linkage) to reduce the risk of false positives arising from population structure. However, *Q*-values alone are not adequate because the *Q*-matrix only provides a rough dissection of population differentiation [22]. More recently, a unified mixed model method, Q+K, that combines information from population structure and relatedness (kinship), has been shown to be superior to these former methods [22], [23]. In the present study, these two statistical models are available for testing associations, the number of significant markers (*P*<0.05) was 15 using GLM but reduced to 13 using MLM (data not shown). This result was consistent with previous studies reporting that MLM was better than GLM at reducing false positive associations. Therefore, we ultimately used MLM to test our phenotype-genotype associations. Similarly, Ehrenreich *et al*. [24] used MLM to conduct candidate gene association mapping of *Arabidopsis* flowering time. In addition, Sexton *et al*. [11] used MLM for association studies in *Eucalyptus pilularis* Smith. Taken together, choosing of the proper statistical models can help to reduce the number of false positive associations.

Validation under either different environmental conditions and/or genetic backgrounds has become the gold standard for assessing statistical results from association studies, even though this replication requirement may cause real genetic effects to be missed [25]. The importance of validation has been well established in candidate gene-based association studies, and several examples in forest trees have recently been published [11], [13], [26]–[28]. The current lack of validation across different association and field-testing environments in this study, and the varying numbers of significant markers (*P*<0.05) using GLM and MLM (data not shown), suggest that these estimates should be considered with caution. To compensate for the lack of a validation population, we conducted functional validation of SNP associations via gene expression analyses to identify whether these significant associations affect gene expression at the mRNA level. And the functional support for significant associations in LD mapping strategy lends strength to their proposed effects.

### Linkage Disequilibrium Test in Trees

Linkage disequilibrium (LD) refers to the nonrandom association of alleles at different loci [29] and plays an important role in association studies for identifying significant markers or haplotypes. Therefore, understanding the patterns of LD in the species is the important prerequisite for association mapping, whether genome-wide associations are feasible or whether a candidate gene-based approach has to be considered. In this study, a rapid LD decay was observed in *PtGA20Ox* (*r* ^2^<0.1, within 500 bp; Figure 3). It is consistent with previous studies that suggested limited LD in trees. For example, Brown *et al.* [30] found a rapid decline in LD within several kilobases in loblolly pine. Similar results of limited LD were reported for candidate genes in other species [13], [26], [31]–[33]. In *Populus*, previous studies based on SNP markers have indicated that a rapid decay of LD occurs within just 300–1,700 bp in candidate genes among related species of *Populus* [15], [34]–[36], suggesting high recombination rates in this outcrossing species. However, Slavov *et al* [37] found that the decay of linkage disequilibrium with physical distance was slower than expected from previous studies in the *P. trichocarpa* genome, with *r^2^* dropping below 0.2 within 3–6 kb.

The reasons for the low LD in forest trees may be a large effective population size, trees’ outcrossing habit and long history of recombination [38]. The low LD usually seen in forest trees suggests that candidate-gene-based LD mapping should be the ideal approach to understand the molecular basis underlying quantitative variation in trees. However, genome-wide association mapping strategies may be inefficient because a large number of markers are needed.

### Detection of Phenotype-genotype Associations in *P.* *tomentosa*

Candidate gene-based LD mapping approaches have been used to dissect complex growth and wood-property traits in forest trees [13], [15], [26]–[28], [39], [40]. Generally, the power of a single-marker association test is often limited because LD information contained in flanking markers is ignored. Intuitively, haplotypes (a block of linked ordered markers) may be more powerful than individual markers [15]. In this study, a comparison of single-marker and haplotype-based association demonstrates that the single-marker-based association analyses have either similar or greater power than haplotype-based tests (Table 2 and 4). These results suggest that single-marker-based tests were preferred to haplotype-based tests to avoid uncertainty in haplotype determination from diploid SNP data sets. Therefore, combining these two methods may provide a better potential to detect functional allelic variance underlying quantitative traits in association populations.

In our study, the fact that significant associations were found with both growth (e.g., tree height) and wood quality (e.g., fiber length, fiber width, microfibril angle and holocellulose content) traits is encouraging (see Table 2), as we are usually interested in selecting for both traits simultaneously. In total, seven significant associations were found and the phenotypic variance explained by a single SNP association ranges from 3.44% to 14.47%. Most of the associations explained a small proportion of the phenotypic variance. This was consistent with other association studies in which between 1.5% and 6.5% of the total phenotypic variation was accounted for by SNPs [13], [26]–[28], [40]. These small SNP effects are in accordance with polygenic quantitative models of wood traits [31]. Among the significant associations, SNP10 explained 14.47% and 10.20% of phenotypic variance in fiber length and width traits, respectively. The effects which were high compared to other studies of quantitative traits in trees may be due to the underlying biological mechanism or other factors. These estimates need to be further validated.

Our discovery of a non-synonymous exonic SNP (SNP10) is somewhat surprising. SNP10 was significantly associated with three wood-quality traits and one growth trait, which may represent pleiotropic effects of the *PtGA20Ox* gene [41]. A similar phenomenon has been identified in previous studies. For example, Beaulieu *et al*. [40] found some SNPs to be significantly associated with more than one trait whereas others were positive with both the additive and the dominant effects models. Similarly, Southerton *et al*. [14] identified two SNPs in *Eni-CAD1* and four SNPs in *Eni-HB1* that were associated with multiple wood traits. SNP10 was located in exon 1 of *PtGA20Ox* and represented a missense mutation. This mutation leads to an amino acid change from Asn to Ser. The traits that associated with SNP10 were fiber length, fiber width, holocellulose content and tree height. The phenomenon that SNP10 was significantly associated with both fiber length and fiber width was consistent with the strong positive phenotypic correlation between these two traits. The association between SNP10 and fiber length was consistent with previous studies that GA alone can affect the length of xylem fibers, as injecting GA inhibitors into woody stems leads to reductions in fiber length [42]. Also, GA-induced increase in both the number and length of xylem fibers in transgenic GA-overproducing trees [43], suggests that GA is mainly active during xylem fiber development. Three significant common haplotypes surrounding SNP10 were also significantly associated with fiber length, and had significant differences (1.2220 mm for C-A-C, 1.1765 mm for A-A-T and 1.1601 mm for A-G-T). Holocellulose is the total of the polysaccharide components of the walls of the secondary xylem cells, which are almost entirely composed of cellulose and hemicelluloses, accounting for nearly 80% of secondary xylem tissue [44]. In the present study, differences in holocellulose content among the three genotypes of SNP10 were significant (74.4160% for AA, 72.5503% for AG and 66.6800% for GG), illustrating that patterns of gene action are consistent with dominant effects. Besides SNP10, six common haplotypes surrounding SNP10 were significantly associated with tree height. The associations with tree height provided evidence for the theory that GAs can accelerate the growth and development of plants. Characterization of GA-deficient mutants has established that GA has a role in promoting shoot growth and stem elongation, as GA mutants are associated with severe dwarfism [45]. GA first promotes cell elongation, and cell division could be stimulated as a result of cell growth [46], [47]. In addition, quantitative Real-time PCR also showed significant expression differences among the three genotypes of SNP10. Moreover, most of the common haplotypes significantly associated with wood traits surround SNP10. Taken together, these results strongly suggest that SNP10 may be a functional polymorphism that is in or near a locus involved in the control of wood traits of *P. tomentosa*. In addition, the four traits (fiber width, fiber length, holocellulose content, and tree height) associated with SNP10 are not negatively correlated (Table S2). Thus, SNP10 could serve as an important marker in breeding for improved growth and wood-property traits of *P. tomentosa*.

SNP19, SNP22 and SNP29 were also significant markers. They were located in different regions of *PtGA20Ox*, including exon, intron, and 3′UTR locations. Silent SNPs should not be considered *a priori* as potential false positives because they may affect transcript levels and codon usage [48], [49]. In this study, SNP19 represented a synonymous substitution that was significantly associated with fiber width. A similar phenomenon has been identified in previous studies. For example, Thumma *et al*. [26] discovered a synonymous exonic SNP of *EniCOBL4A* associated with cellulose content and kraft pulp yield. Similarly, Southerton *et al*. [50] confirmed that the CO07 SNP in *Eni-COBL4A* was a synonymous substitution but was significantly associated with cellulose content. Both SNP22 located in intron 2 and SNP29 located in 3′UTR occurred in non-coding regions of *PtGA20Ox*. SNPs in introns could affect phenotypic traits because those particular introns may play an important role in regulating gene expression and exon splicing. Although the mutation of the 3′ flanking region did not result in an amino acid change, it may regulate expression of the gene. Previous studies have observed that sequences in the 3′ flanking region can affect the mechanisms of mRNA deadenylation and degradation [51], [52]. Significant SNP markers in non-coding regions of a gene, such as introns and 3′UTR, have also been reported elsewhere. Gonzalez-Martinez *et al*. [33] analyzed association genetics in *Pinus taeda* and found a strong association between SNP M10 located in intron 1 and earlywood microfibril angle. Fang *et al*. [53] detected a novel SNP in the 3′ flanking region of the goat *BMP-2 *gene associated with growth traits.

These association results suggest that *PtGA20Ox* can affect the growth and development, fiber formation, and wood quality of *P. tomentosa*; these findings are consistent with previous studies. In terms of tree breeding, *GA20Ox* has been found to affect the growth and wood quality of trees. Eriksson *et al*. [43] transferred *GA20Ox* of *Arabidopsis thaliana* (*AtGA20Ox*) to *P*. *tremula* × *P*. *tremuloides* and discovered higher levels of endogenous GA and improved biomass with bigger leaves, a taller stem, and more fiber cells in the xylem. This suggested that *PtGA20Ox* may have a similar function considering that the *PtGA20Ox* protein shares 65.8% identity with AtGA20Ox. Similarly, Deng *et al*. [54] transferred cotton *GA20Ox* to *P. tomentosa* and found that stem growth and biomass were enhanced. Thus, *PtGA20Ox* is an important candidate gene for future tree-breeding programs. The SNP markers identified in this study can be applied to breeding programs. However, a combination of these significant markers may be required because the percentage of trait variation explained by each individual SNP was small.

## Materials and Methods

### Association Population

The association population consisted of 426 unrelated *P. tomentosa* individuals growing in Guan Xian County, Shandong Province, where root segments of 1047 native individuals collected from the entire natural distribution range of *P*. *tomentosa* were used to establish a clonal arboretum in 1982 using a randomized complete block design with three replications. On the basis of principal component analysis and ISODATA fuzzy clustering of 16 meteorological factors [19], the total climatic zone covered by these individuals can be divided into three large climatic regions: southern, northwestern, and northeastern. In the present study, 426 unrelated individuals representing almost the entire geographic distribution of *P. tomentosa* (180 from the southern region, 86 from the northwestern region, and 160 from the northeastern region) were used for association analysis. In addition, 36 *P. tomentosa* individuals were sequenced to identify SNPs within *PtGA20Ox*.

This study was carried out in strict accordance with the recommendations in the Guide for Observational and field studies. All necessary permits were obtained for the described field studies. The sampling of all individuals of *P*. *tomentosa* was approved by the Youhui Zhang, director of National Garden of *P*. *tomentosa* in Guan Xian County, Shandong Province.

### Phenotypic Data

Ten traits were measured: lignose content, holocellulose content, alpha-cellulose content, fiber length, fiber width, microfibril angle, tree height, tree diameter, volume of wood, and tree height/tree diameter. In this study, wood sample materials containing bark and pith were cored from each of the 426 poplars in the association population at breast height using increment borers. These sample materials (15 cm long × 10 mm in diameter) were collected in 2010.

Four referential standard procedures (GB/T2677.8–1994, GB/T2677.10–1995, GB/T 744–2004, and FZ/T50010.4–1998) were consulted to test for contents of lignose, holocellulose, and alpha-cellulose, with consideration of the experimental conditions. Fiber length and width were measured using a Colour CCTV Camera (Panasonic SDII), and microfibril angle was measured using an X-ray powder diffractometer (Philips). Data for tree height and diameter at breast height were collected during field surveys in 2011; these data were used to determine the volume of wood. SAS for Windows ver. 8.2 (SAS Institute, Cary, NC, USA) was used to conduct analysis of variance (ANOVA) and correlations for the above phenotypic traits.

### Isolation of *PtGA20Ox* cDNA

The *P*. *tomentosa* stem mature xylem cDNA library was constructed using the Superscript λ System following the manufacturer’s suggestions (Life Technologies, Rockville, MD, USA). The constructed cDNA library consisted of 5.0 × 10^6^ pfu with an insert size of 1.0–4.0 kb. Random end sequencing of 500 cDNA clones and comparison with all available *Arabidopsis GA20Ox* sequences identified a full-length cDNA with high similarity to *AtGA20Ox*; this was named *PtGA20Ox*.

### SNP Identification

To identify SNPs, 36 unrelated individuals from the association population were used to sequence *GA20Ox*. First, genomic DNA (20 ng per reaction) isolated separately from the 36 unrelated individuals of the association population was used as an amplification template to clone the *GA20Ox* gene using gene specific primers. The primer pairs for amplification were designed using Primer 3 software (primer3.sourceforge.net). All PCR products were resolved by agarose gel electrophoresis, excised, and purified using Ultrafree®-DA (Millipore, Billerica, MA, USA) centrifugal filter units. The purified DNA was then ligated into the pGEM®-T Easy Vector and transformed into JM109 competent cells (Promega). Plasmid DNA was extracted from overnight cultures using the QIAprep Spin Miniprep protocol and was sequenced on both strands with conservative primers using the Big Dye Terminator version 3.1 Cycle Sequencing kit (Applied Biosystems, Beijing, China) and a LI-COR 4300 genetic analyzer. Finally, the 36 genomic clones were sequenced and analyzed using the software MEGA4.0 and DnaSP4.50.4.

### SNP Genotyping

Twenty-nine common SNPs were genotyped in 426 trees in the association population by amplification using locked nucleic acid (LNA) technology [55], [56]. Amplification was performed in a final reaction volume of 25 µl containing 20 ng genomic DNA, 0.8 U *Taq* DNA polymerase (Promega), 50 ng forward primer, 50 ng reverse primer, 10×PCR buffer (Promega), and 0.2 mM dNTPs (Promega). The PCR conditions were as follows: 94°C for 3 min, 30 cycles of 94°C denaturation for 30 s, annealing at 54–58°C (depending on the primers) for 15 s, and extension at 72°C for 1 min, with a final extension at 72°C for 5 min.

### Real-time Quantitative PCR

Real-time quantitative PCR was performed on a DNA Engine Opticon 2 machine (MJ Research) using the LightCycler-FastStar DNA master SYBR Green I kit (Roche). The PCR program included an initial denaturation at 94°C for 5 min; 40 cycles of 30 s at 94°C, 30 s at 58°C, and 30 s at 72°C; and a final melt-curve of 70–95°C. The specificity of the amplified fragments was checked using the generated melting curve. All reactions were conducted in triplicate, and the generated real-time data were analyzed using the Opticon Monitor Analysis Software 3.1 tool.

### Data Analysis

Linkage disequilibrium analysis: We used “SAS genetics” to test Hardy-Weinberg equilibrium (HWE) of the SNPs and estimated the relationship of linkage disequilibrium with physical distance by using the linear regression analysis approach which is built into the DnaSP software, version 4.0 [57], [58]. The software package HAPLOVIEW (http://www.broad.mit.edu/mpg/haploview.html) [59] was used to evaluate LD among 29 SNPs in *P. tomentosa*. The *r^2^* (squared allele frequency) is the parameter most frequently used to estimate LD [60]–[62]. The interval value of the parameter varies from 0 to 1. The significance (*P*-values) of *r^2^* for each SNP locus was calculated using 100,000 permutations.

Association testing: The phenotype-genotype associations in this study were identified by comparing results from a general linear model (GLM) and a mixed linear model (MLM) in the software package TASSEL2.1 (http://www2.maizegenetics.net/index.php?page=bioinformatics/tassel/index.html) [21], [63]. The values of estimated membership probability (*Q*) and pairwise kinship (*K*) were used to evaluate the effects of population structure and relatedness among individuals for marker-trait associations. The GLM only uses the *Q* value, whereas MLM uses both *Q* and *K* values. The *Q* matrix was identified based on the significant subpopulations (K = 11) [20] by 20 neutral genomic SSR markers, as assessed according to the statistical model described by [64], using the software package STRUCTURE VERISON 2.3 (http://pritch.bsd.uchicago.edu/structure.html) [65]. The K matrix was calculated on the basis of 20 SSR loci using the method proposed by Ritland [66], which is built into the program SPAGeDi, version 1.3 [67]. The K matrix was calculated as described by Yu *et al*. [21] and all negative values between individuals were set to 0. The false positive discovery rate (FDR) method was additionally applied to correct for multiple testing using QVALUE software [68], [69].

Haplotype analysis: Haplotype frequencies from genotype data were estimated, and haplotype association tests were conducted on a three-marker sliding window using haplotype trend regression software [70]. The significance of the haplotype associations was based on 1000 permutation tests.

Modes of gene action: The modes of gene action were quantified using the ratio of dominance (d) to additive (a) effects estimated from least-square means for each genotypic class. Partial or complete dominance was defined as values in the range 0.50<|d/a|<1.25, whereas additive effects were defined as values in the range |d/a|≤0.5. Values of |d/a|>1.25 were equated with under- or overdominance [15], [71].

## Supporting Information

Table S1 The minimum, and maximum values, mean, standard error (SE) and coefficient of phenotypic variation [CV (%)] for each growth and wood property trait measured in *P. tomentosa* association population.
(DOC)

Table S2 Estimates of phenotypic correlations (*R*) for these ten phenotypic traits in the association population.
(DOC)

Table S3 All the association results. a. All the association results using the general linear model (GLM). b. All the association results using the mixed linear model (MLM).
(DOC)
