# Supplementary material for: Allelic Variation in PtGA20Ox Associates with Growth and Wood Properties in Populus spp
Source: PLoS One. 2012 Dec 31;7(12):e53116. doi: 10.1371/journal.pone.0053116 (PMC3534044; doi:10.1371/journal.pone.0053116)
Supplement: Table S1 — The minimum, and maximum values, mean, standard error (SE) and coefficient of phenotypic variation [CV (%)] for each growth and wood property trait measured in P. tomentosa association population. (DOC) [file pone.0053116.s001.doc]

**Table S1.** The minimum, and maximum values, mean, standard error (SE) and coefficient of phenotypic variation [CV (%)] for each growth and wood property trait measured in *P. tomentosa* association population.

| Statistics | Lignin  (%) | Holocellulose  (%) | α-cellulose  (%) | Fiber length  (mm) | Fiber width  (µm) | MFA  (deg) | D  (cm) | H  (m) | V  (m^3^ ) | H/D |
| --- | --- | --- | --- | --- | --- | --- | --- | --- | --- | --- |
| Min | 21.53 | 64.13 | 40.63 | 0.866 | 16.984 | 15.22 | 8.46 | 2.90 | 0.037 | 0.065 |
| Max | 28.68 | 87.40 | 47.74 | 1.512 | 33.503 | 24.78 | 44.43 | 22.50 | 3.022 | 2.660 |
| Mean | 23.85 | 73.58 | 44. 53 | 1.170 | 23.160 | 18.23 | 21.45 | 14.61 | 0.602 | 1.503 |
| SE | 0.002 | 0.032 | 0.015 | 4.070 ×10^-3^ | 0.096 | 0.027 | 0.072 | 0.018 | 0.360×10^-3^ | 5.684×10^-3^ |
| CV (%) | 3.59 | 5.05 | 5.77 | 7.15 | 8.56 | 19.30 | 26.80 | 19.90 | 67.60 | 21.04 |

*H* tree height, *D* the diameter at breast height, *V* stem volume, *MFA* microfiber angle, *N* number of trees sampled.
