# Supplementary material for: Allelic Variation in PtGA20Ox Associates with Growth and Wood Properties in Populus spp
Source: PLoS One. 2012 Dec 31;7(12):e53116. doi: 10.1371/journal.pone.0053116 (PMC3534044; doi:10.1371/journal.pone.0053116)
Supplement: Table S2 — Estimates of phenotypic correlations (R) for these ten phenotypic traits in the association population. (DOC) [file pone.0053116.s002.doc]

**Table S2.** Estimates of phenotypic correlations (*R*) for these ten phenotypic traits in the association population.

| Traits | Lignin | Holocellulose | α-cellulose | Fiber length | Fiber width | MFA | D | H | V | H/D |
| --- | --- | --- | --- | --- | --- | --- | --- | --- | --- | --- |
| Lignin | 1 |  |  |  |  |  |  |  |  |  |
| Holocellulose | -0.225** | 1 |  |  |  |  |  |  |  |  |
| α-cellulose | -0.256** | 0.993** | 1 |  |  |  |  |  |  |  |
| Fiber length | 0.040 | 0.041 | 0.036 | 1 |  |  |  |  |  |  |
| Fiber width | 0.083 | 0.076 | 0.078 | 0.200** | 1 |  |  |  |  |  |
| MFA | -0.003 | -0.001 | -0.004 | -0.080 | -0.014 | 1 |  |  |  |  |
| D | 0.001 | 0.005 | 0.006 | 0.044 | -0.001 | 0.006 | 1 |  |  |  |
| H | 0.013 | 0.008 | -0.001 | 0.277** | 0.013 | -0.018 | -0.015 | 1 |  |  |
| V | 0.000187 | 0.005 | 0.006 | 0.042 | -0.002 | 0.007 | 1.000** | -0.019 | 1 |  |
| H/D | -0.066 | -0.038 | -0.044 | -0.117 | -0.217** | 0.007 | -0.236** | 0.126** | -0.232** | 1 |

H = tree height, D *=* the diameter at breast height, V = stem volume, MFA *=* microfiber angle, ** *P*<0.01.
