# Supplementary material for: Allelic Variation in PtGA20Ox Associates with Growth and Wood Properties in Populus spp
Source: PLoS One. 2012 Dec 31;7(12):e53116. doi: 10.1371/journal.pone.0053116 (PMC3534044; doi:10.1371/journal.pone.0053116)
Supplement: Table S3 — All the association results. a. All the association results using the general linear model (GLM). b. All the association results using the mixed linear model (MLM). (DOC) [file pone.0053116.s003.doc]

**Table S3** All the association results.

1. All the association results using the general linear model (GLM).

| Trait | Locus | P-value | R^2^ |
| --- | --- | --- | --- |
| lignin content | S01 | 0.6638 | 0.0021 |
| lignin content | S02 | 0.6404 | 0.0022 |
| lignin content | S03 | 0.0935 | 0.0119 |
| lignin content | S04 | 0.4732 | 0.0038 |
| lignin content | S05 | 0.4538 | 0.004 |
| lignin content | S06 | 0.8942 | 5.65E-04 |
| lignin content | s07 | 0.5625 | 0.0029 |
| lignin content | s08 | 0.1249 | 0.0105 |
| lignin content | s09 | 0.6393 | 5.54E-04 |
| lignin content | s10 | 0.2585 | 0.0068 |
| lignin content | s11 | 0.0614 | 0.014 |
| lignin content | s12 | 0.2176 | 0.0077 |
| lignin content | s13 | 0.2176 | 0.0077 |
| lignin content | s14 | 0.7875 | 0.0012 |
| lignin content | s15 | 0.7875 | 0.0012 |
| lignin content | s16 | 0.359 | 0.0052 |
| lignin content | s17 | 0.2519 | 0.0033 |
| lignin content | s18 | 0.6876 | 4.08E-04 |
| lignin content | s19 | 0.901 | 3.91E-05 |
| lignin content | s20 | 0.2294 | 0.0036 |
| lignin content | s21 | 0.358 | 0.0021 |
| lignin content | s22 | 0.7293 | 0.0016 |
| lignin content | s23 | 0.1977 | 0.0082 |
| lignin content | s24 | 0.3349 | 0.0055 |
| lignin content | s25 | 0.5787 | 0.0028 |
| lignin content | s26 | 0.1024 | 0.0114 |
| lignin content | s27 | 0.9293 | 3.76E-04 |
| lignin content | s28 | 0.4612 | 0.0039 |
| lignin content | s29 | 0.0295 | 0.0179 |
| holocellulose content | S01 | 0.8618 | 7.26E-04 |
| holocellulose content | S02 | 0.8139 | 0.001 |
| holocellulose content | S03 | 0.3596 | 0.005 |
| holocellulose content | S04 | 0.0775 | 0.0124 |
| holocellulose content | S05 | 0.3677 | 0.0049 |
| holocellulose content | S06 | 0.0289 | 0.0171 |
| holocellulose content | s07 | 0.8203 | 9.64E-04 |
| holocellulose content | s08 | 0.0465 | 0.0148 |
| holocellulose content | s09 | 0.7387 | 2.71E-04 |
| holocellulose content | s10 | 2.87E-04 | 0.039 |
| holocellulose content | s11 | 0.0811 | 0.0122 |
| holocellulose content | s12 | 0.8322 | 8.94E-04 |
| holocellulose content | s13 | 0.8322 | 8.94E-04 |
| holocellulose content | s14 | 0.6454 | 0.0021 |
| holocellulose content | s15 | 0.6454 | 0.0021 |
| holocellulose content | s16 | 0.6544 | 0.0021 |
| holocellulose content | s17 | 0.5892 | 7.09E-04 |
| holocellulose content | s18 | 0.5551 | 8.46E-04 |
| holocellulose content | s19 | 0.9495 | 9.75E-06 |
| holocellulose content | s20 | 0.9522 | 8.75E-06 |
| holocellulose content | s21 | 0.7052 | 3.48E-04 |
| holocellulose content | s22 | 0.1492 | 0.0092 |
| holocellulose content | s23 | 0.9496 | 2.52E-04 |
| holocellulose content | s24 | 0.704 | 0.0017 |
| holocellulose content | s25 | 0.5969 | 0.0025 |
| holocellulose content | s26 | 0.1758 | 0.0084 |
| holocellulose content | s27 | 0.1787 | 0.0085 |
| holocellulose content | s28 | 0.6469 | 0.0021 |
| holocellulose content | s29 | 0.5497 | 0.003 |
| α-cellulose content | S01 | 0.5262 | 0.0032 |
| α-cellulose content | S02 | 0.9319 | 3.48E-04 |
| α-cellulose content | S03 | 0.1869 | 0.0082 |
| α-cellulose content | S04 | 0.3036 | 0.0059 |
| α-cellulose content | S05 | 0.1812 | 0.0084 |
| α-cellulose content | S06 | 0.3009 | 0.0059 |
| α-cellulose content | s07 | 0.9474 | 2.67E-04 |
| α-cellulose content | s08 | 0.0492 | 0.0147 |
| α-cellulose content | s09 | 0.7967 | 1.64E-04 |
| α-cellulose content | s10 | 0.064 | 0.0135 |
| α-cellulose content | s11 | 0.5989 | 0.0025 |
| α-cellulose content | s12 | 0.7696 | 0.0013 |
| α-cellulose content | s13 | 0.7696 | 0.0013 |
| α-cellulose content | s14 | 0.6812 | 0.0019 |
| α-cellulose content | s15 | 0.6812 | 0.0019 |
| α-cellulose content | s16 | 0.7405 | 0.0015 |
| α-cellulose content | s17 | 0.3615 | 0.002 |
| α-cellulose content | s18 | 0.8009 | 1.57E-04 |
| α-cellulose content | s19 | 0.7304 | 2.92E-04 |
| α-cellulose content | s20 | 0.9738 | 2.66E-06 |
| α-cellulose content | s21 | 0.985 | 8.75E-07 |
| α-cellulose content | s22 | 0.7253 | 0.0016 |
| α-cellulose content | s23 | 0.1273 | 0.0101 |
| α-cellulose content | s24 | 0.604 | 0.0025 |
| α-cellulose content | s25 | 0.0959 | 0.0115 |
| α-cellulose content | s26 | 0.7491 | 0.0014 |
| α-cellulose content | s27 | 0.1502 | 0.0094 |
| α-cellulose content | s28 | 0.8043 | 0.0011 |
| α-cellulose content | s29 | 0.2054 | 0.0079 |
| fiber length | S01 | 0.867 | 6.81E-04 |
| fiber length | S02 | 0.8275 | 9.02E-04 |
| fiber length | S03 | 0.8715 | 6.55E-04 |
| fiber length | S04 | 0.7403 | 0.0014 |
| fiber length | S05 | 0.6845 | 0.0018 |
| fiber length | S06 | 0.8757 | 6.32E-04 |
| fiber length | s07 | 0.8466 | 7.92E-04 |
| fiber length | s08 | 0.5752 | 0.0026 |
| fiber length | s09 | 0.7342 | 2.74E-04 |
| fiber length | s10 | 1.00E-07 | 0.0739 |
| fiber length | s11 | 0.9058 | 4.71E-04 |
| fiber length | s12 | 0.4121 | 0.0042 |
| fiber length | s13 | 0.4121 | 0.0042 |
| fiber length | s14 | 0.3263 | 0.0053 |
| fiber length | s15 | 0.3263 | 0.0053 |
| fiber length | s16 | 0.2176 | 0.0072 |
| fiber length | s17 | 0.9902 | 3.56E-07 |
| fiber length | s18 | 0.9177 | 2.54E-05 |
| fiber length | s19 | 0.0896 | 0.0068 |
| fiber length | s20 | 0.9307 | 1.80E-05 |
| fiber length | s21 | 0.6253 | 5.67E-04 |
| fiber length | s22 | 0.5842 | 0.0026 |
| fiber length | s23 | 0.0729 | 0.0124 |
| fiber length | s24 | 0.5754 | 0.0026 |
| fiber length | s25 | 0.3996 | 0.0044 |
| fiber length | s26 | 0.5239 | 0.0031 |
| fiber length | s27 | 0.069 | 0.0128 |
| fiber length | s28 | 0.9676 | 1.57E-04 |
| fiber length | s29 | 0.8874 | 5.79E-04 |
| fiber width | S01 | 0.9553 | 2.20E-04 |
| fiber width | S02 | 0.6961 | 0.0017 |
| fiber width | S03 | 0.0144 | 0.0202 |
| fiber width | S04 | 0.8901 | 5.60E-04 |
| fiber width | S05 | 0.4997 | 0.0033 |
| fiber width | S06 | 0.9748 | 1.23E-04 |
| fiber width | s07 | 0.8272 | 9.11E-04 |
| fiber width | s08 | 0.3341 | 0.0053 |
| fiber width | s09 | 0.2809 | 0.0028 |
| fiber width | s10 | 2.78E-08 | 0.0803 |
| fiber width | s11 | 0.303 | 0.0057 |
| fiber width | s12 | 0.982 | 8.73E-05 |
| fiber width | s13 | 0.982 | 8.73E-05 |
| fiber width | s14 | 0.5308 | 0.003 |
| fiber width | s15 | 0.5308 | 0.003 |
| fiber width | s16 | 0.9254 | 3.73E-04 |
| fiber width | s17 | 0.4746 | 0.0012 |
| fiber width | s18 | 0.284 | 0.0028 |
| fiber width | s19 | 0.7655 | 2.13E-04 |
| fiber width | s20 | 0.7149 | 3.20E-04 |
| fiber width | s21 | 0.8853 | 5.00E-05 |
| fiber width | s22 | 2.08E-04 | 0.0399 |
| fiber width | s23 | 0.2825 | 0.0061 |
| fiber width | s24 | 0.7261 | 0.0015 |
| fiber width | s25 | 0.8787 | 6.21E-04 |
| fiber width | s26 | 0.0542 | 0.0139 |
| fiber width | s27 | 0.1531 | 0.0091 |
| fiber width | s28 | 0.8342 | 8.71E-04 |
| fiber width | s29 | 0.5723 | 0.0027 |
| microfiber angle | S01 | 0.543 | 0.0029 |
| microfiber angle | S02 | 0.6952 | 0.0017 |
| microfiber angle | S03 | 0.259 | 0.0064 |
| microfiber angle | S04 | 0.5148 | 0.0031 |
| microfiber angle | S05 | 0.5671 | 0.0027 |
| microfiber angle | S06 | 0.8797 | 6.09E-04 |
| microfiber angle | s07 | 0.8566 | 7.35E-04 |
| microfiber angle | s08 | 0.9736 | 1.27E-04 |
| microfiber angle | s09 | 0.8823 | 5.20E-05 |
| microfiber angle | s10 | 0.1497 | 0.009 |
| microfiber angle | s11 | 0.2436 | 0.0067 |
| microfiber angle | s12 | 0.547 | 0.0029 |
| microfiber angle | s13 | 0.547 | 0.0029 |
| microfiber angle | s14 | 0.7654 | 0.0013 |
| microfiber angle | s15 | 0.7654 | 0.0013 |
| microfiber angle | s16 | 0.5575 | 0.0028 |
| microfiber angle | s17 | 0.3058 | 0.0025 |
| microfiber angle | s18 | 0.7609 | 2.20E-04 |
| microfiber angle | s19 | 0.8749 | 5.89E-05 |
| microfiber angle | s20 | 0.006 | 0.0178 |
| microfiber angle | s21 | 0.0423 | 0.0097 |
| microfiber angle | s22 | 0.5758 | 0.0026 |
| microfiber angle | s23 | 0.7439 | 0.0014 |
| microfiber angle | s24 | 0.8456 | 7.96E-04 |
| microfiber angle | s25 | 0.3515 | 0.005 |
| microfiber angle | s26 | 0.5587 | 0.0028 |
| microfiber angle | s27 | 0.2629 | 0.0064 |
| microfiber angle | s28 | 0.3108 | 0.0055 |
| microfiber angle | s29 | 0.8819 | 6.08E-04 |
| D | S01 | 0.9857 | 6.70E-05 |
| D | S02 | 0.8965 | 5.07E-04 |
| D | S03 | 0.9808 | 8.97E-05 |
| D | S04 | 0.9939 | 2.82E-05 |
| D | S05 | 0.9811 | 8.83E-05 |
| D | S06 | 0.989 | 5.12E-05 |
| D | s07 | 0.9426 | 2.74E-04 |
| D | s08 | 0.9856 | 6.71E-05 |
| D | s09 | 0.6408 | 5.04E-04 |
| D | s10 | 0.9656 | 1.63E-04 |
| D | s11 | 0.9863 | 6.40E-05 |
| D | s12 | 0.8919 | 5.30E-04 |
| D | s13 | 0.8919 | 5.30E-04 |
| D | s14 | 0.7941 | 0.0011 |
| D | s15 | 0.7941 | 0.0011 |
| D | s16 | 0.9651 | 1.65E-04 |
| D | s17 | 0.98 | 1.45E-06 |
| D | s18 | 0.9267 | 1.96E-05 |
| D | s19 | 0.9891 | 4.35E-07 |
| D | s20 | 0.6446 | 4.93E-04 |
| D | s21 | 0.8204 | 1.19E-04 |
| D | s22 | 0.9927 | 3.42E-05 |
| D | s23 | 0.9698 | 1.42E-04 |
| D | s24 | 0.9979 | 9.62E-06 |
| D | s25 | 0.9944 | 2.62E-05 |
| D | s26 | 0.8263 | 8.85E-04 |
| D | s27 | 0.9864 | 6.44E-05 |
| D | s28 | 0.9712 | 1.36E-04 |
| D | s29 | 0.7238 | 0.0015 |
| H | S01 | 0.5886 | 0.0024 |
| H | S02 | 0.4832 | 0.0033 |
| H | S03 | 0.8338 | 8.19E-04 |
| H | S04 | 0.0436 | 0.014 |
| H | S05 | 0.3246 | 0.0051 |
| H | S06 | 0.9217 | 3.67E-04 |
| H | s07 | 0.5261 | 0.0029 |
| H | s08 | 0.1236 | 0.0094 |
| H | s09 | 0.3863 | 0.0017 |
| H | s10 | 0.0023 | 0.0271 |
| H | s11 | 0.6881 | 0.0017 |
| H | s12 | 0.7705 | 0.0012 |
| H | s13 | 0.7705 | 0.0012 |
| H | s14 | 0.9211 | 3.71E-04 |
| H | s15 | 0.9211 | 3.71E-04 |
| H | s16 | 0.307 | 0.0053 |
| H | s17 | 0.7416 | 2.45E-04 |
| H | s18 | 0.9594 | 5.83E-06 |
| H | s19 | 0.7059 | 3.21E-04 |
| H | s20 | 0.2291 | 0.0032 |
| H | s21 | 0.4135 | 0.0015 |
| H | s22 | 0.6263 | 0.0021 |
| H | s23 | 0.7113 | 0.0015 |
| H | s24 | 0.6349 | 0.002 |
| H | s25 | 0.2938 | 0.0055 |
| H | s26 | 0.0523 | 0.0132 |
| H | s27 | 0.8053 | 9.92E-04 |
| H | s28 | 0.3059 | 0.0053 |
| H | s29 | 0.2463 | 0.0064 |
| V | S01 | 0.985 | 7.04E-05 |
| V | S02 | 0.8934 | 5.23E-04 |
| V | S03 | 0.9818 | 8.52E-05 |
| V | S04 | 0.9933 | 3.10E-05 |
| V | S05 | 0.9821 | 8.38E-05 |
| V | S06 | 0.9887 | 5.29E-05 |
| V | s07 | 0.9388 | 2.93E-04 |
| V | s08 | 0.9853 | 6.87E-05 |
| V | s09 | 0.6369 | 5.16E-04 |
| V | s10 | 0.9737 | 1.24E-04 |
| V | s11 | 0.9847 | 7.15E-05 |
| V | s12 | 0.8912 | 5.34E-04 |
| V | s13 | 0.8912 | 5.34E-04 |
| V | s14 | 0.7897 | 0.0011 |
| V | s15 | 0.7897 | 0.0011 |
| V | s16 | 0.9677 | 1.53E-04 |
| V | s17 | 0.982 | 1.18E-06 |
| V | s18 | 0.9256 | 2.02E-05 |
| V | s19 | 0.9879 | 5.30E-07 |
| V | s20 | 0.6543 | 4.64E-04 |
| V | s21 | 0.8275 | 1.10E-04 |
| V | s22 | 0.9923 | 3.58E-05 |
| V | s23 | 0.9706 | 1.38E-04 |
| V | s24 | 0.998 | 9.52E-06 |
| V | s25 | 0.9944 | 2.63E-05 |
| V | s26 | 0.8307 | 8.60E-04 |
| V | s27 | 0.9871 | 6.10E-05 |
| V | s28 | 0.9708 | 1.37E-04 |
| V | s29 | 0.7243 | 0.0015 |
| H/D | S01 | 0.4613 | 0.0033 |
| H/D | S02 | 0.9745 | 1.11E-04 |
| H/D | S03 | 0.6067 | 0.0021 |
| H/D | S04 | 0.7094 | 0.0015 |
| H/D | S05 | 0.0773 | 0.0109 |
| H/D | S06 | 0.4234 | 0.0037 |
| H/D | s07 | 0.2471 | 0.006 |
| H/D | s08 | 0.1099 | 0.0094 |
| H/D | s09 | 0.9827 | 1.00E-06 |
| H/D | s10 | 0.004 | 0.0234 |
| H/D | s11 | 0.1842 | 0.0072 |
| H/D | s12 | 0.6261 | 0.002 |
| H/D | s13 | 0.6261 | 0.002 |
| H/D | s14 | 0.3226 | 0.0048 |
| H/D | s15 | 0.3226 | 0.0048 |
| H/D | s16 | 0.0777 | 0.0109 |
| H/D | s17 | 0.7011 | 3.15E-04 |
| H/D | s18 | 0.9577 | 6.02E-06 |
| H/D | s19 | 0.824 | 1.06E-04 |
| H/D | s20 | 0.0127 | 0.0132 |
| H/D | s21 | 0.1006 | 0.0058 |
| H/D | s22 | 0.7876 | 0.001 |
| H/D | s23 | 0.5172 | 0.0028 |
| H/D | s24 | 0.5827 | 0.0023 |
| H/D | s25 | 0.2513 | 0.0059 |
| H/D | s26 | 0.6588 | 0.0018 |
| H/D | s27 | 0.6975 | 0.0016 |
| H/D | s28 | 0.9973 | 1.17E-05 |
| H/D | s29 | 0.6216 | 0.0021 |

1. All the association results using the mixed linear model (MLM).

| Trait | Locus | P-value | R^2^ |
| --- | --- | --- | --- |
| lignin content | S01 | 0.7011 | 0.0023 |
| lignin content | S02 | 0.6488 | 0.0027 |
| lignin content | S03 | 0.1435 | 0.0123 |
| lignin content | S04 | 0.3874 | 0.006 |
| lignin content | S05 | 0.5997 | 0.0032 |
| lignin content | S06 | 0.8176 | 0.0013 |
| lignin content | s07 | 0.5377 | 0.0039 |
| lignin content | s08 | 0.1807 | 0.0108 |
| lignin content | s09 | 0.4904 | 0.0015 |
| lignin content | s10 | 0.0965 | 0.0148 |
| lignin content | s11 | 0.9948 | 1.33E-07 |
| lignin content | s12 | 0.4013 | 0.0058 |
| lignin content | s13 | 0.4013 | 0.0058 |
| lignin content | s14 | 0.8251 | 0.0012 |
| lignin content | s15 | 0.8251 | 0.0012 |
| lignin content | s16 | 0.3523 | 0.0066 |
| lignin content | s17 | 0.2494 | 0.0042 |
| lignin content | s18 | 0.5569 | 0.0011 |
| lignin content | s19 | 0.4543 | 0.005 |
| lignin content | s20 | 0.1997 | 0.0052 |
| lignin content | s21 | 0.8317 | 1.43E-04 |
| lignin content | s22 | 0.3874 | 0.006 |
| lignin content | s23 | 0.7093 | 0.0022 |
| lignin content | s24 | 0.479 | 0.0047 |
| lignin content | s25 | 0.4031 | 0.0058 |
| lignin content | s26 | 0.1182 | 0.0135 |
| lignin content | s27 | 0.9586 | 2.71E-04 |
| lignin content | s28 | 0.3078 | 0.0075 |
| lignin content | s29 | 0.4924 | 0.0015 |
| holocellulose content | S01 | 0.8855 | 7.36E-04 |
| holocellulose content | S02 | 0.9845 | 9.44E-05 |
| holocellulose content | S03 | 0.3836 | 0.0058 |
| holocellulose content | S04 | 0.1003 | 0.0138 |
| holocellulose content | S05 | 0.6151 | 0.0029 |
| holocellulose content | S06 | 0.0619 | 0.0167 |
| holocellulose content | s07 | 0.8511 | 9.73E-04 |
| holocellulose content | s08 | 0.068 | 0.0161 |
| holocellulose content | s09 | 0.6167 | 7.55E-04 |
| holocellulose content | s10 | 5.61E-05 | 0.0578 |
| holocellulose content | s11 | 0.8578 | 9.69E-05 |
| holocellulose content | s12 | 0.9634 | 2.25E-04 |
| holocellulose content | s13 | 0.9634 | 2.25E-04 |
| holocellulose content | s14 | 0.7335 | 0.0019 |
| holocellulose content | s15 | 0.7335 | 0.0019 |
| holocellulose content | s16 | 0.6731 | 0.0024 |
| holocellulose content | s17 | 0.5261 | 0.0012 |
| holocellulose content | s18 | 0.8394 | 1.24E-04 |
| holocellulose content | s19 | 0.8087 | 0.0013 |
| holocellulose content | s20 | 0.9797 | 1.96E-06 |
| holocellulose content | s21 | 0.7969 | 2.00E-04 |
| holocellulose content | s22 | 0.968 | 1.96E-04 |
| holocellulose content | s23 | 0.2249 | 0.009 |
| holocellulose content | s24 | 0.8915 | 6.93E-04 |
| holocellulose content | s25 | 0.5339 | 0.0038 |
| holocellulose content | s26 | 0.139 | 0.0119 |
| holocellulose content | s27 | 0.3858 | 0.0058 |
| holocellulose content | s28 | 0.8884 | 7.29E-04 |
| holocellulose content | s29 | 0.5953 | 8.50E-04 |
| α-cellulose content | S01 | 0.5975 | 0.003 |
| α-cellulose content | S02 | 0.5401 | 0.0036 |
| α-cellulose content | S03 | 0.1835 | 0.0098 |
| α-cellulose content | S04 | 0.2071 | 0.0091 |
| α-cellulose content | S05 | 0.1545 | 0.0108 |
| α-cellulose content | S06 | 0.5487 | 0.0035 |
| α-cellulose content | s07 | 0.9806 | 1.13E-04 |
| α-cellulose content | s08 | 0.0999 | 0.0133 |
| α-cellulose content | s09 | 0.5134 | 0.0012 |
| α-cellulose content | s10 | 0.1111 | 0.0127 |
| α-cellulose content | s11 | 0.8356 | 1.25E-04 |
| α-cellulose content | s12 | 0.8382 | 0.001 |
| α-cellulose content | s13 | 0.8382 | 0.001 |
| α-cellulose content | s14 | 0.7569 | 0.0016 |
| α-cellulose content | s15 | 0.7569 | 0.0016 |
| α-cellulose content | s16 | 0.7794 | 0.0014 |
| α-cellulose content | s17 | 0.3344 | 0.0027 |
| α-cellulose content | s18 | 0.9564 | 8.66E-06 |
| α-cellulose content | s19 | 0.4246 | 0.005 |
| α-cellulose content | s20 | 0.8404 | 1.18E-04 |
| α-cellulose content | s21 | 0.6188 | 7.17E-04 |
| α-cellulose content | s22 | 0.2022 | 0.0092 |
| α-cellulose content | s23 | 0.8684 | 8.19E-04 |
| α-cellulose content | s24 | 0.8177 | 0.0012 |
| α-cellulose content | s25 | 0.0949 | 0.0136 |
| α-cellulose content | s26 | 0.8216 | 0.0011 |
| α-cellulose content | s27 | 0.1092 | 0.013 |
| α-cellulose content | s28 | 0.6705 | 0.0024 |
| α-cellulose content | s29 | 0.6037 | 7.80E-04 |
| fiber length | S01 | 0.6624 | 0.0021 |
| fiber length | S02 | 0.661 | 0.0021 |
| fiber length | S03 | 0.9057 | 5.12E-04 |
| fiber length | S04 | 0.6678 | 0.0021 |
| fiber length | S05 | 0.8462 | 8.62E-04 |
| fiber length | S06 | 0.6778 | 0.002 |
| fiber length | s07 | 0.6259 | 0.0024 |
| fiber length | s08 | 0.6984 | 0.0019 |
| fiber length | s09 | 0.7717 | 2.17E-04 |
| fiber length | s10 | 8.72E-14 | 0.1447 |
| fiber length | s11 | 0.0865 | 0.0076 |
| fiber length | s12 | 0.84 | 9.00E-04 |
| fiber length | s13 | 0.84 | 9.00E-04 |
| fiber length | s14 | 0.2284 | 0.0076 |
| fiber length | s15 | 0.2284 | 0.0076 |
| fiber length | s16 | 0.0899 | 0.0124 |
| fiber length | s17 | 0.5621 | 8.67E-04 |
| fiber length | s18 | 0.934 | 1.77E-05 |
| fiber length | s19 | 9.94E-05 | 0.0466 |
| fiber length | s20 | 0.6433 | 5.53E-04 |
| fiber length | s21 | 0.6179 | 6.42E-04 |
| fiber length | s22 | 0.0623 | 0.0142 |
| fiber length | s23 | 0.5699 | 0.0029 |
| fiber length | s24 | 0.3137 | 0.006 |
| fiber length | s25 | 0.5144 | 0.0034 |
| fiber length | s26 | 0.4447 | 0.0042 |
| fiber length | s27 | 0.1265 | 0.0107 |
| fiber length | s28 | 0.0102 | 0.0176 |
| fiber length | s29 | 0.6463 | 5.43E-04 |
| fiber width | S01 | 0.986 | 7.79E-05 |
| fiber width | S02 | 0.5689 | 0.0031 |
| fiber width | S03 | 0.026 | 0.02 |
| fiber width | S04 | 0.885 | 6.76E-04 |
| fiber width | S05 | 0.6518 | 0.0024 |
| fiber width | S06 | 0.9622 | 2.13E-04 |
| fiber width | s07 | 0.9652 | 1.96E-04 |
| fiber width | s08 | 0.2447 | 0.0078 |
| fiber width | s09 | 0.5261 | 0.0011 |
| fiber width | s10 | 4.17E-09 | 0.102 |
| fiber width | s11 | 0.793 | 1.90E-04 |
| fiber width | s12 | 0.4543 | 0.0044 |
| fiber width | s13 | 0.4543 | 0.0044 |
| fiber width | s14 | 0.8595 | 8.37E-04 |
| fiber width | s15 | 0.8595 | 8.37E-04 |
| fiber width | s16 | 0.8341 | 0.001 |
| fiber width | s17 | 0.8258 | 1.34E-04 |
| fiber width | s18 | 0.287 | 0.0031 |
| fiber width | s19 | 0.0836 | 0.0136 |
| fiber width | s20 | 0.437 | 0.0017 |
| fiber width | s21 | 0.989 | 5.25E-07 |
| fiber width | s22 | 4.98E-04 | 0.0413 |
| fiber width | s23 | 0.2224 | 0.0083 |
| fiber width | s24 | 0.8768 | 7.26E-04 |
| fiber width | s25 | 0.7383 | 0.0017 |
| fiber width | s26 | 0.0457 | 0.0169 |
| fiber width | s27 | 0.149 | 0.0106 |
| fiber width | s28 | 0.7128 | 0.0019 |
| fiber width | s29 | 0.7794 | 2.17E-04 |
| microfiber angle | S01 | 0.523 | 0.0035 |
| microfiber angle | S02 | 0.4999 | 0.0037 |
| microfiber angle | S03 | 0.3259 | 0.006 |
| microfiber angle | S04 | 0.6832 | 0.002 |
| microfiber angle | S05 | 0.3508 | 0.0056 |
| microfiber angle | S06 | 0.7045 | 0.0019 |
| microfiber angle | s07 | 0.6743 | 0.0021 |
| microfiber angle | s08 | 0.8562 | 8.33E-04 |
| microfiber angle | s09 | 0.8849 | 5.62E-05 |
| microfiber angle | s10 | 0.1335 | 0.0108 |
| microfiber angle | s11 | 0.8404 | 1.09E-04 |
| microfiber angle | s12 | 0.6838 | 0.002 |
| microfiber angle | s13 | 0.6838 | 0.002 |
| microfiber angle | s14 | 0.8275 | 0.001 |
| microfiber angle | s15 | 0.8275 | 0.001 |
| microfiber angle | s16 | 0.7089 | 0.0018 |
| microfiber angle | s17 | 0.2512 | 0.0035 |
| microfiber angle | s18 | 0.6515 | 5.47E-04 |
| microfiber angle | s19 | 0.914 | 4.83E-04 |
| microfiber angle | s20 | 0.9219 | 2.58E-05 |
| microfiber angle | s21 | 0.0777 | 0.0083 |
| microfiber angle | s22 | 0.7889 | 0.0013 |
| microfiber angle | s23 | 0.4314 | 0.0045 |
| microfiber angle | s24 | 0.8785 | 6.95E-04 |
| microfiber angle | s25 | 0.2066 | 0.0084 |
| microfiber angle | s26 | 0.4626 | 0.0041 |
| microfiber angle | s27 | 0.2252 | 0.008 |
| microfiber angle | s28 | 0.213 | 0.0084 |
| microfiber angle | s29 | 0.0033 | 0.0436 |
| D | S01 | 0.9931 | 3.68E-05 |
| D | S02 | 0.9522 | 2.62E-04 |
| D | S03 | 0.9984 | 8.54E-06 |
| D | S04 | 0.9113 | 4.96E-04 |
| D | S05 | 0.9911 | 4.77E-05 |
| D | S06 | 0.9787 | 1.15E-04 |
| D | s07 | 0.9892 | 5.77E-05 |
| D | s08 | 0.9998 | 8.30E-07 |
| D | s09 | 0.4945 | 0.0012 |
| D | s10 | 0.8656 | 7.72E-04 |
| D | s11 | 0.8773 | 6.35E-05 |
| D | s12 | 0.9374 | 3.45E-04 |
| D | s13 | 0.9374 | 3.45E-04 |
| D | s14 | 0.8927 | 6.05E-04 |
| D | s15 | 0.8927 | 6.05E-04 |
| D | s16 | 0.9853 | 7.92E-05 |
| D | s17 | 0.9618 | 6.12E-06 |
| D | s18 | 0.823 | 1.33E-04 |
| D | s19 | 0.8315 | 9.85E-04 |
| D | s20 | 0.9944 | 1.33E-07 |
| D | s21 | 0.716 | 3.53E-04 |
| D | s22 | 0.9488 | 2.80E-04 |
| D | s23 | 0.9986 | 7.72E-06 |
| D | s24 | 0.9928 | 3.87E-05 |
| D | s25 | 1 | 1.47E-07 |
| D | s26 | 0.8044 | 0.0012 |
| D | s27 | 0.9918 | 4.49E-05 |
| D | s28 | 0.7303 | 0.0017 |
| D | s29 | 0.7407 | 2.92E-04 |
| H | S01 | 0.5673 | 0.0029 |
| H | S02 | 0.2495 | 0.007 |
| H | S03 | 0.8873 | 6.03E-04 |
| H | S04 | 0.0207 | 0.0194 |
| H | S05 | 0.353 | 0.0052 |
| H | S06 | 0.9555 | 2.29E-04 |
| H | s07 | 0.3737 | 0.005 |
| H | s08 | 0.0522 | 0.0148 |
| H | s09 | 0.3656 | 0.0021 |
| H | s10 | 9.89E-04 | 0.0344 |
| H | s11 | 0.6595 | 4.89E-04 |
| H | s12 | 0.9274 | 3.80E-04 |
| H | s13 | 0.9274 | 3.80E-04 |
| H | s14 | 0.9517 | 2.50E-04 |
| H | s15 | 0.9517 | 2.50E-04 |
| H | s16 | 0.1904 | 0.0083 |
| H | s17 | 0.8021 | 1.58E-04 |
| H | s18 | 0.8282 | 1.19E-04 |
| H | s19 | 0.5438 | 0.0031 |
| H | s20 | 0.6082 | 6.62E-04 |
| H | s21 | 0.106 | 0.0066 |
| H | s22 | 0.8036 | 0.0011 |
| H | s23 | 0.5756 | 0.0028 |
| H | s24 | 0.5624 | 0.0029 |
| H | s25 | 0.1544 | 0.0094 |
| H | s26 | 0.1079 | 0.0112 |
| H | s27 | 0.8953 | 5.69E-04 |
| H | s28 | 0.0162 | 0.0209 |
| H | s29 | 0.0775 | 0.0078 |
| V | S01 | 0.9937 | 3.39E-05 |
| V | S02 | 0.9491 | 2.79E-04 |
| V | S03 | 0.9985 | 8.04E-06 |
| V | S04 | 0.9078 | 5.16E-04 |
| V | S05 | 0.9911 | 4.75E-05 |
| V | S06 | 0.9781 | 1.18E-04 |
| V | s07 | 0.9874 | 6.79E-05 |
| V | s08 | 0.9995 | 2.72E-06 |
| V | s09 | 0.4904 | 0.0013 |
| V | s10 | 0.8787 | 6.92E-04 |
| V | s11 | 0.8775 | 6.33E-05 |
| V | s12 | 0.94 | 3.30E-04 |
| V | s13 | 0.94 | 3.30E-04 |
| V | s14 | 0.8868 | 6.41E-04 |
| V | s15 | 0.8868 | 6.41E-04 |
| V | s16 | 0.9864 | 7.33E-05 |
| V | s17 | 0.9594 | 6.92E-06 |
| V | s18 | 0.8237 | 1.32E-04 |
| V | s19 | 0.8325 | 9.79E-04 |
| V | s20 | 0.995 | 1.06E-07 |
| V | s21 | 0.7074 | 3.76E-04 |
| V | s22 | 0.9492 | 2.79E-04 |
| V | s23 | 0.9984 | 8.50E-06 |
| V | s24 | 0.9927 | 3.92E-05 |
| V | s25 | 1 | 2.24E-07 |
| V | s26 | 0.8076 | 0.0011 |
| V | s27 | 0.9916 | 4.55E-05 |
| V | s28 | 0.7291 | 0.0017 |
| V | s29 | 0.7301 | 3.17E-04 |
| H/D | S01 | 0.5757 | 0.0027 |
| H/D | S02 | 0.7872 | 0.0012 |
| H/D | S03 | 0.8666 | 7.07E-04 |
| H/D | S04 | 0.5691 | 0.0028 |
| H/D | S05 | 0.1804 | 0.0084 |
| H/D | S06 | 0.6902 | 0.0018 |
| H/D | s07 | 0.3281 | 0.0055 |
| H/D | s08 | 0.1511 | 0.0093 |
| H/D | s09 | 0.8533 | 8.44E-05 |
| H/D | s10 | 0.0467 | 0.0151 |
| H/D | s11 | 0.6713 | 4.44E-04 |
| H/D | s12 | 0.7218 | 0.0016 |
| H/D | s13 | 0.7218 | 0.0016 |
| H/D | s14 | 0.0612 | 0.0137 |
| H/D | s15 | 0.0612 | 0.0137 |
| H/D | s16 | 0.0804 | 0.0124 |
| H/D | s17 | 0.4895 | 0.0012 |
| H/D | s18 | 0.8937 | 4.41E-05 |
| H/D | s19 | 0.3141 | 0.0057 |
| H/D | s20 | 0.4548 | 0.0014 |
| H/D | s21 | 0.2877 | 0.0028 |
| H/D | s22 | 0.3666 | 0.0049 |
| H/D | s23 | 0.6953 | 0.0018 |
| H/D | s24 | 0.5701 | 0.0028 |
| H/D | s25 | 0.0938 | 0.0116 |
| H/D | s26 | 0.6601 | 0.0021 |
| H/D | s27 | 0.923 | 4.02E-04 |
| H/D | s28 | 0.392 | 0.0047 |
| H/D | s29 | 0.0852 | 0.0073 |

H = tree height, D *=* the diameter at breast height, V = stem volume.
